# Cut-off values of serum IgG4 among three reagents, including a novel IgG4 reagent: a multicenter study

**DOI:** 10.1038/s41598-021-86024-5

**Published:** 2021-03-31

**Authors:** Yoko Usami, Mitsutoshi Sugano, Takeshi Uehara, Masayoshi Koinuma, Nau Ishimine, Kenji Kawasaki, Kazuyoshi Yamauchi, Hideaki Hamano, Takayuki Honda

**Affiliations:** 1 Department of Laboratory Medicine, Shinshu University Hospital, Matsumoto, Japan; 2 Department of Laboratory Medicine, Shinshu University School of Medicine, 3-1-1 Asahi, Matsumoto, 390-8621 Japan; 3 Faculty of Pharmaceutical Sciences, Teikyo Heisei University, Tokyo, Japan; 4 Center of Clinical Research, Shinshu University Hospital, Matsumoto, Japan; 5 Department of Laboratory Medicine, Chiba University School of Medicine, Chiba, Japan; 6 Department of Laboratory Medicine, Faculty of Medicine, University of Tsukuba, Tsukuba, Japan; 7 Nagano Prefectural Kiso Hospital, Kiso, Japan

**Keywords:** Biomarkers, Biomarkers

## Abstract

Elevated serum IgG4 is a useful marker of IgG4-related disease (IgG4-RD) activity. However, there is no uniformity in the cut-off values of IgG4 among the various reagents. The aim of this study was to compare the measured and cut-off values of IgG4 assessed using three different reagents. This study enrolled 466 IgG4-RD and non-IgG4-RD patients who required measurement of serum IgG4 levels to diagnose or treat IgG4-RD. Serum IgG4 was measured using three reagents: N-assay LA IgG4 Nittobo (Nittobo), BS-NIA IgG4 (TBS), and N Latex IgG4 (Siemens). The values obtained using the three reagents were compared, and cut-off values were calculated for each. Although there was good correlation among the results with the three reagents, the measured and cut-off values were all different. The Nittobo values were 1.4 times the TBS values and the TBS values were almost half those of the Siemens values. ROC curve analysis showed cut-off values for the Nittobo, TBS, and Siemens reagents of 1.42, 1.31, and 2.38 g/L, respectively. The measured and cut-off values of serum IgG4 vary depending on the reagents used for the assay, although there is good correlation among the values measured by the three reagents.

## Introduction

IgG4-related disease (IgG4-RD) is a new disease recognized in this century and is characterized by the formation of masses or nodules/hypertrophic lesions in various organs. Prominent lymphocyte and IgG4-positive plasma cell infiltration^1^ and characteristic fibrosis, called storiform fibrosis, are observed at the site of the lesion^2^. The disease is particularly frequent in middle-aged and elderly men and is characterized by significant response to steroid treatment^3^. Although fibro-inflammation with elevated serum IgG4 was first recognized in autoimmune pancreatitis (AIP), it has since been found to affect almost all organs and is conceptualized as a group of diseases^4^.

Although the mechanisms of the onset of IgG4-RD have not been identified, involvement of certain autoantibodies has been implicated. Patients with AIP are known to have various autoantibodies, such as antinuclear antibodies, anti-carbonic anhydrase-II (CA-II) antibodies, anti-lactoferrin antibodies, rheumatoid factor and anti-smooth muscle antibodies. Although these autoantibodies have low disease specificity, certain new disease-specific autoantibodies have been found. Shiokawa et al. reported that IgG4 and IgG1 in the serum of IgG4-RD patients cause pancreatic injury, and that the antigen for the antibody in AIP patients was laminin 511^5,6^. At the same time, Hubers et al. reported that the antigen for autoantibodies in AIP patients was annexin A11^7^. Other reports have shown that the expression level of galectine-3 is high in IgG4-RD patients, and that anti-galectine-3 antibody titers increase in proportion to plasma galectine-3 levels^8,9^. However, while these autoantibodies have been found in some cases, their further verification as disease-specific antigens is desired in the future.

Elevated serum IgG4 is one of the hallmarks of IgG4-RD, and is a useful marker for diagnosis, follow-up and monitoring for recurrence. The reported cut-off value of 1.35 g/L was calculated using a comparison between pancreatic cancer and AIP patients^10^. Serum IgG4 measurement reagents are supplied by two companies, The Binding Site (TBS, Birmingham, UK) and Siemens Healthcare Diagnostics (Siemens, Eschborn, Germany). In addition, a new reagent has recently been released by Nittobo Medical Co., Ltd. (Nittobo, Tokyo, Japan) and has begun to be used in Japan. However, all three reagents result in different measured values^11^. This is reflected in the differences in their reference ranges: the upper limit of the reference range for the Siemens reagent is about twice that of the TBS reagent. A report on the cut-off value for IgG4-RD from China stated a value of 2.48 g/L, which is about twice the diagnostic standard of 1.35 g/L in Japan^12^. This is not due to differences in research subjects, but to differences in the reagents used.

Although there are many reports on calculation of the cut-off value for a single reagent, no reports have shown how the cut-off values differ between reagents. In this study, we report the results of simultaneous measurement of IgG4-RD in patient samples using reagents from the three companies, and calculation of the cut-off values for each reagent.

## Results

### Characteristics of patients and serum IgG4 levels

We grouped the 466 enrolled patients according to their diagnosis and treatment. All patients who had received some kind of treatment, including medications such as steroids and surgical treatment at the time of blood collection, were grouped as “during or post-treatment”. The median serum IgG4 levels in each group are shown in Table 1. Three hundred forty-two patients (73%) were diagnosed with IgG4-RD (definite IgG4-RD group), 56 of whom were untreated at the time of blood collection and the remaining 286 of whom were evaluated during or after treatment. Thirty-six patients (8%) were suspected to have IgG4-RD but were not confirmed (suspicious IgG4-RD group), and the remaining 88 patients (19%) were diagnosed or suspected to have non-IgG4-RD. In the definite and suspicious IgG4-RD groups, 70% of patients were male, while 40% of patients in the non-IgG4-RD group were male. Median IgG4 levels in pre-treatment patients were significantly higher than those in during or post-treatment patients in the definite and suspicious IgG4-RD group, although there was no significant difference between the pretreatment and treatment subgroups in the non-IgG4-RD group. The same trend was observed for all three reagents. Comparing the median and interquartile range (IQR) of IgG4 levels measured with the three reagents, the values were in ascending order with TBS, Nittobo and Siemens reagents, except that values were similar with Nittobo and TBS reagents in the non-IgG4-RD group.

**Table 1:** Characteristics of patients and median serum IgG4 levels with each reagent.

	N	Male/Female (%)	Age		IgG4 (g/L)								
					NBM			TBS			Siemens		
			Median	IQR	Median	IQR	*P*	Median	IQR	*P*	Median	IQR	*P*
All patients	466	299/64 (64/36)	69	62–75	2.24	0.87–4.71		1.91	0.83–3.84		3.34	1.28–6.49	
**Definite IgG4-RD**													
Pre-treatment	56	33/23 (59/41)	68	57–76	6.20	2.64–9.98	< 0.001	4.63	2.16–7.50	< 0.001	9.06	3.75–13.8	< 0.001
During or post- treatment	286	206/80 (72/28)	70	63–75	2.51	1.35–4.68		2.12	1.18–3.84		3.70	2.04–6.48	
**Suspicious IgG4-RD**													
Pre-treatment	22	16/6 (73/27)	66	64–74	4.40	2.35–7.43	< 0.001	3.65	2.00–5.46	< 0.001	5.66	3.71–10.7	< 0.001
During or post- treatment	14	10/4 (71/29)	69	66–76	1.50	1.06–1.82		1.31	0.92–1.71		2.07	1.57–2.95	
**Non-IgG4-RD**													
Pre-treatment	54	26/28 (48/52)	68	58–74	0.46	0.24–0.73	0.706	0.39	0.23–0.61	0.787	0.52	0.25–0.91	0.668
During or post- treatment	34	8/26 (24/76)	68	61–73	0.39	0.21–0.87		0.34	0.17–0.80		0.47	0.16–1.28	

*IQR* interquartile range.

### Correlations between the three reagents

**Figure 1:**
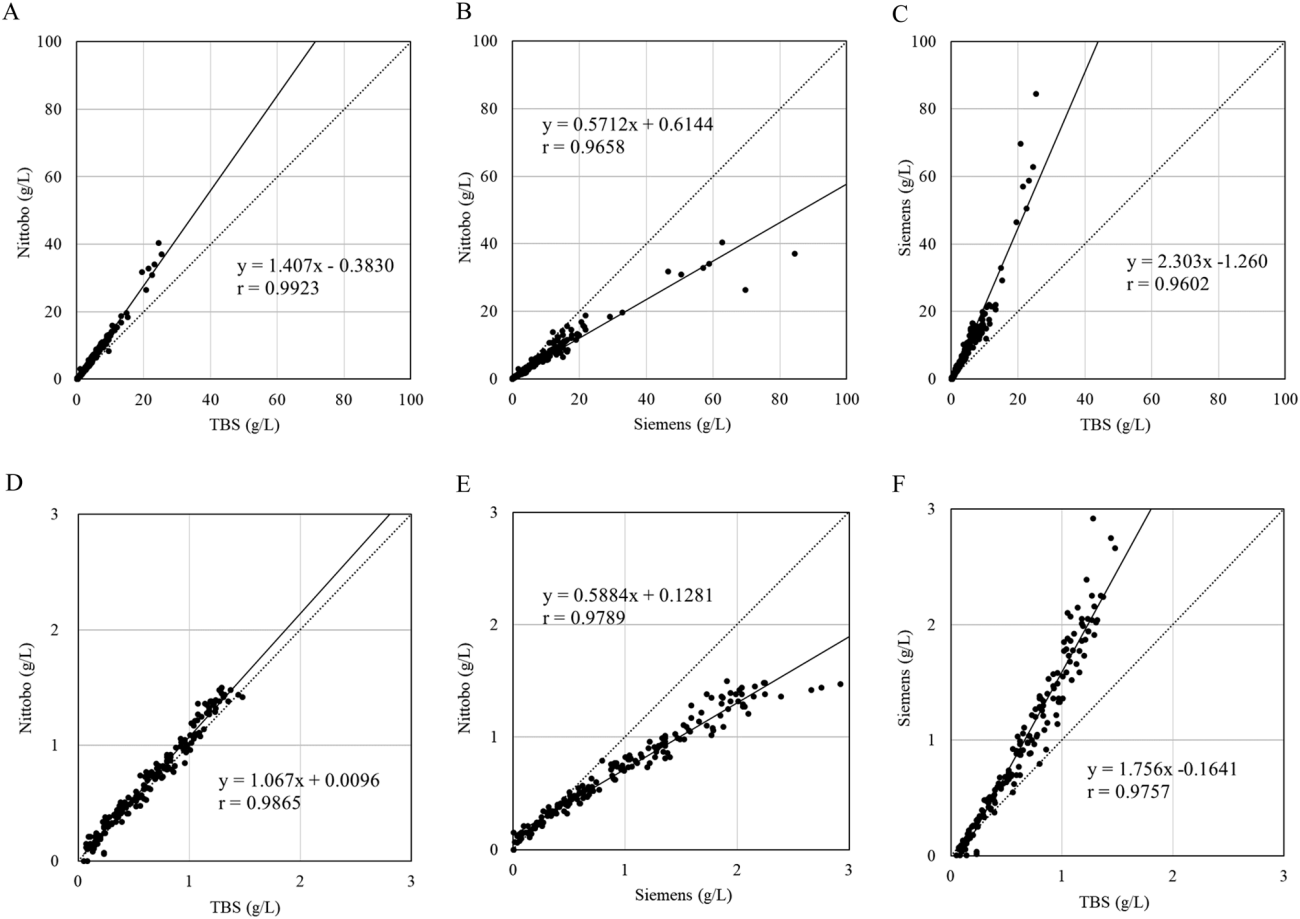
Correlations between the three reagents. Correlation diagrams for the three reagents using all patient data are shown in panels (**A**), (**B**) and (**C**). Panels (**D**), (**E**) and (**F**) show the data of patients with Nittobo values less than 1.5 g/L. Regression lines were calculated using major-axis linear regression and drawn as the solid line together with the small dotted line of equality (Y = X).

Correlations between the three reagents (Nittobo, TBS and Siemens) for all 466 patients are shown in Fig. 1A,B,C. For all combinations of comparisons, the major-axis regression lines deviated from the diagonal line of equality (broken line). This indicated that the values measured by the three reagents were all different. However, when the analysis was limited to patients with IgG4 levels less than 1.5 g/L (Nittobo value) (Fig. 1D,E,F), the range included almost all healthy individuals, and the values obtained with Nittobo and TBS matched perfectly (Fig. 1D). In comparisons between Nittobo and Siemens, the slope of the regression line did not change in comparisons between all patients and for patients with IgG4 values below 1.5 g/L (Fig. 1B,E), with the values measured by Nittobo being almost 0.6 times lower than the value obtained with the Siemens reagent. In the comparison of IgG4 values measured by TBS and Siemens reagents, although the slope of the regression line was slightly different when comparing all patients versus patients with values below 1.5 g/L, the value with TBS was almost half that of the value with Siemens.

### Cut-off values for each reagent

**Figure 2:**
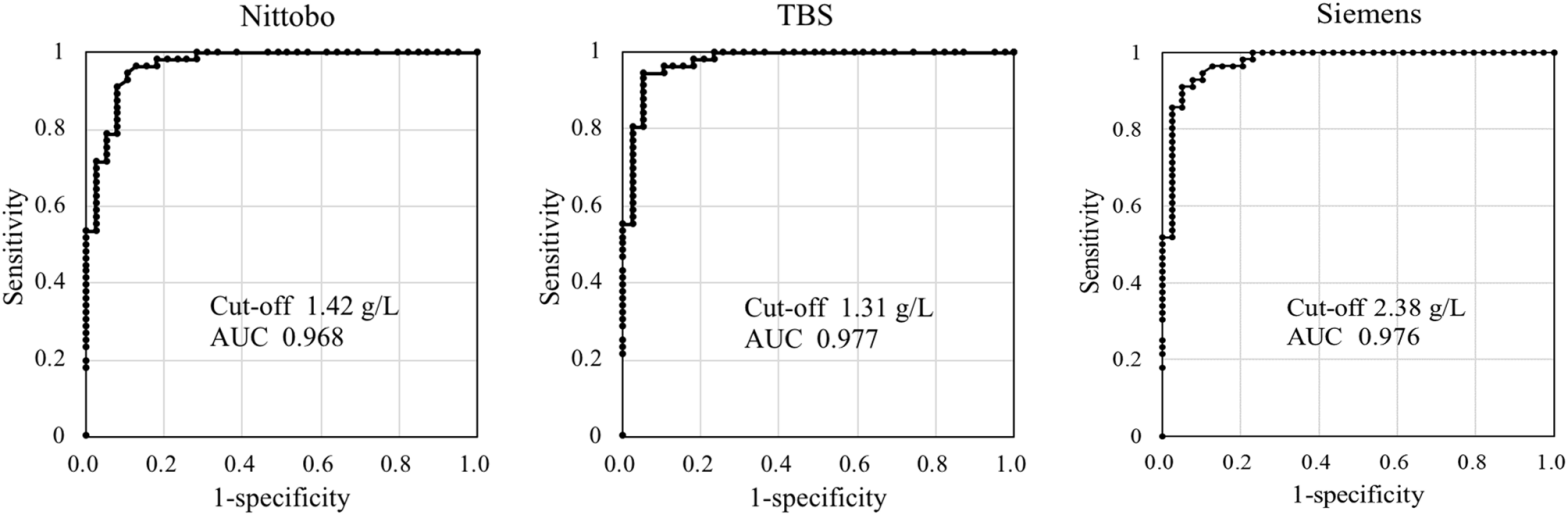
Receiver operating characteristic (ROC) curve analysis of IgG4 levels measured using the three reagents. The area under the ROC curve and cut-off value for each reagent were calculated using the data of the definite IgG4-RD group (n = 56) and untreated and diagnosed non-IgG4-RD patients from the non-IgG4-RD group (n = 41).

ROC analysis was performed between untreated patients in the definite IgG4-RD group (n = 56) and untreated and diagnosed with non-IgG4-RD patients in the non-IgG4-RD group (n = 41). Pre-treatment patients in the non-IgG4-RD group (n = 54) included both patients with confirmed non-IgG4-RD disease and those with suspected non-IgG4-RD disease. ROC analysis used data from only patients with confirmed non-IgG4-RD (n = 41). The involved organs in IgG4-RD patients and the diseases in non-IgG4-RD patients are shown in Table 2. Thirty-four patients in the IgG4-RD group had lesions in only a single organ, while 22 patients had lesions in more than one organ. Fifty-two patients (93%) had lesions in the lacrimal gland, salivary gland and/or pancreas. The most common disease in the non-IgG4-RD group was pancreatic cancer, followed by Sjögren syndrome and pancreatitis. The ROC curve, cut-off value and area under the curve (AUC), sensitivity and specificity for each reagent are shown in Fig. 2 and Table 3. The cut-off value of Nittobo was 1.42 g/L, which was close to the cut-off value of 1.31 g/L of TBS, although the cut-off value of Siemens was nearly double. Although the cut-off values differed among the three reagents, no significant differences in AUC, sensitivity, and specificity were observed.

**Table 2:** Organs involved in the IgG4-RD group and diseases in the non-IgG4-RD group.

Involved organ	n	%
**IgG4-RD (n = 56)**		
Lacrimal gland, salivary gland	34	60.7
Pancreas	27	48.2
Retroperitoneum	5	8.9
Lung	4	7.1
Bile duct	4	7.1
Kidney	3	5.4
Hypophysis	1	1.8
Lymph node	1	1.8
Other	1	1.8

Disease	n	%
**Non-IgG4-RD (n = 41)**		
Pancreatic cancer	9	22.0
Sjögren syndrome	8	19.5
Pancreatitis	7	17.1
Tumor-forming pancreatitis	2	4.9
Intraductal papillary mucinous tumor	2	4.9
Pancreatic neuroendocrine tumor	2	4.9
Abdominal aortic aneurysm	2	4.9
Cholangiocarcinoma	1	2.4
Carcinoma of the papilla of Vater	1	2.4
Stomach cancer	1	2.4
Ovarian cancer	1	2.4
Lymphoma	1	2.4
Primary sclerosing cholangitis	1	2.4
Hepatitis	1	2.4
Basedow disease	1	2.4
Stenosis of the bile duct	1	2.4

ROC analysis was performed between 56 pre-treatment patients in the definite IgG4-RD group and 41 patients with confirmed non-IgG4-RD disease in pre-treatment patient of the non-IgG4-RD group.

**Table 3:** Cut-off value and AUC for each reagent.

	Cut-off (g/L)	AUC	Sensitivity	Specificity
Nittobo	1.42	0.968	0.946	0.897
TBS	1.31	0.977	0.946	0.949
Siemens	2.38	0.976	0.911	0.949

*AUC* area under ROC curve.

## Discussion

In this study, we evaluated correlations between three IgG4 reagents using 466 patients’ samples, and calculated the cut-off value for each reagent. The correlation between the three reagents was compared over a wide range of values and patients, including not only IgG4-RD patients, but also patients with other diseases that need to be distinguished from IgG4-RD. The results showed that although there was good correlation between the results with the three reagents, the measured values were all different. As a result, the cut-off values also varied depending on the reagent used. This is the first study to simultaneously calculate the IgG4-RD cut-off value for the three IgG4 reagents.

The cut-off value of 1.31 g/L of the TBS reagent in this study was almost the same as the value of 1.35 g/L determined using the TBS reagent in 2001^10^. The calculated cut-off value of 1.41 g/L for Nittobo was very close to that of TBS, and the slope of the regression equation of the correlation for values less than 1.5 g/L was 1.0. Therefore, a cut-off value of 1.35 g/L can be applied for the Nittobo reagent. On the other hand, a different cut-off value needs to be set for the Siemens reagent. This study calculated a cut-off value of 2.38 g/L for the Siemens reagent, which is consistent with the cut-off value of 2.48 g/L calculated by Yu et al. using nearly 3,000 samples tested with the Siemens reagent^12^.

Diagnosis of IgG4-RD might sometimes be difficult. This is because patients’ characteristics differ depending on the affected organ, and because elevated serum IgG4 levels are not always observed in IgG4-RD patients^13^. Therefore, it is necessary to make a final diagnosis by a combination of evaluations, such as computed tomography, magnetic resonance imaging, and biopsy. However, as evidenced by the high AUC values in the ROC analysis of this study, it is certain that many patients with IgG4-RD are identified by the presence of high serum IgG4 levels, and serum IgG4 levels contribute to the diagnosis of IgG4-RD. Moreover, it has the advantage of being easier to perform than other examinations. However, the differences in measured values between IgG4 assay kits can mislead clinicians when making a diagnosis. The cut-off value for serum IgG4 was described as 1.35 g/L in the Japanese comprehensive diagnostic criteria published in 2011, but this value could be used only for the TBS reagents used in Japan at the time. American College of Rheumatology/European League Against Rheumatism classification criteria for IgG4-RD adopted the upper limit of the reference range of each assay kit as the cut-off level for diagnosis^14^. Since several reagents are used in the United States, it is considered that the reference value of each reagent should be used instead of a specific number. Other measurement kits might become available in different countries in the future. To avoid confusion, harmonization of the measurement values between the assay kits should be achieved.

Differences in IgG subclass values between TBS and Siemens reagents have been previously reported^15^. Although harmonization of the value of total IgG has been achieved using Certified Reference Material (CRM) 470 released by the International Federation of Clinical Chemistry and Laboratory Medicine (IFCC) in 1993, CRM 470 has no certified values for IgG subclasses and the measurements have not been unified. TBS uses CRM 470^16^ and Siemens uses WHO67/97 as the reference material^17^, IgG4 values of each reference material were determined by different researchers. Nittobo has the original master calibrator with IgG4 value determined themselves, and they trace the value to the kit’s calibrator^11^. Those mean that all three companies have established the different traceability. Consequently, the difference in the slope of the correlation results below 1.5 g/L between Siemens and the other two kits could be induced by the difference in the determined values for their own calibrators. According to the report of Ladwig et al., the quantitative value of IgG subclasses measured using LC–MS/MS are consistent with the values measured using the TBS reagent^18^. Bernasconi et al. reported that the amount of purified IgG4 protein was consistent with the value of IgG4 measured using the TBS reagent^19^. These reports suggest that the IgG4 value measured using the TBS reagent is an accurate value.

However, the different calibrators, assessed by the original traceability of each company, cannot explain the mismatch in the slopes of the regression equations between the correlation diagrams for all data and those for the data less than 1.5 g/L in the comparisons between TBS and other two methods. This would mean that even if all methods indicate sufficient linearity in the localized range such as less than 1.5 g/L, no absolute linearity in wide range, such as from 0 to 20 ~ 40 g/L, is provided by all or some of those methods although apparently sufficient linearity is observed in three methods. Therefore, a data around cut off value, which is practically important to diagnose IgG4-RD, is convertible using a constant factor among three methods. However, although the good correlation coefficient for all data was also observed among three methods, it does not mean to be able to convert a data obtained by each method using the same factor described above, especially in a data much higher than cut off value. Imoto et al. reported that the sum of the values of the four IgG subclasses differs from the measured value of IgG when IgG4 levels increase^20^. In healthy individuals with a normal balance of the four subclasses, the total IgG value and sum of the subclass values that is measured using the TBS measurement kit are the same, but divergence occurs when IgG4 is high. From our result, it is considered that the divergence reported by Imoto et al. is due to poor linearity in the high value region in IgG4 measurement. Further validation of the accuracy of the measurement value with elevated IgG4 levels will be needed.

In conclusion, serum IgG4 is a useful marker for IgG4-RD diagnosis and treatment, but the values vary greatly depending on the reagents used for the assay. Therefore, it is necessary to change the cut-off value according to the reagent used. In previous studies, the cut-off value was calculated for only a single reagent, but in this study, cut-off values for three reagents were calculated simultaneously. Any reagents described here are available to diagnose IgG4-RD if the suitable cut-off value for each reagent is used. However, it is disclosed that the proportional conversion for all of the data is not always possible. Therefore, when using serum IgG4 values for the diagnosis and treatment of IgG4-RD, it is essential to confirm which reagent is used. It is hoped that harmonization of IgG4 measurements will be achieved in the near future.

## Methods

### Subjects

This prospective study included not only patients with IgG4-related disease, but also those with differential diseases. Furthermore, we targeted patients who required serum IgG4 measurement for diagnosis or treatment regardless of the presence or absence of a definitive diagnosis and the stage of treatment. We enrolled 466 patients from Sep 2016 to Jan 2017 at 23 hospitals in Japan. The diagnosis of IgG4-RD was made according to the comprehensive diagnostic criteria for IgG4-RD, 2011 (21). The study was conducted according to the guidelines of the Declaration of Helsinki and was approved by the institutional review board of Shinshu University School of Medicine (Matsumoto, Japan) (approval no. 3325). Written informed consent was obtained from all subjects.

### Laboratory analysis

At each hospital, blood that was collected from the subjects was partitioned, and the separated serum was immediately frozen. All serum samples were sent to the clinical laboratory of Shinshu University Hospital and stored at -30 °C until measurement. Serum IgG4 levels in all samples were measured using three reagents, N-assay LA IgG4 Nittobo (Nittobo reagent; Nittobo Medical Co., Ltd., Tokyo, Japan), BS-NIA IgG4 (TBS reagent; The Binding Site, Birmingham, UK) and N Latex IgG4 (Siemens reagent; Siemens Healthcare Diagnostics Products GmbH, Eschborn, Germany). The Nittobo reagent was used with the JCA-BM6070 automatic biochemical analyzer (Japan Electron Optics Laboratory, Tokyo, Japan), and the TBS and Siemens reagents were used with the BN-II nephelometer (Siemens Healthcare Diagnostics Products GmbH, Eschborn, Germany). All assays were performed at our laboratory according to the respective manufacturer’s instructions.

### Statistical analysis

Groups were compared by the Mann–Whitney U test using StatFlex version 6 and those with a P value of less than 0.05 were considered statistically significant. The ROC analysis was performed using JMP version 10, and cut-off values were identified by the Youden index.
